# Pulmonary artery‐focused contrast echocardiography with supplemental oxygen(PCESO) for echocardiographic diagnosis of anomalous origin of left coronary artery from pulmonary artery: Novel use of an old technique

**DOI:** 10.1002/ccr3.2354

**Published:** 2019-08-13

**Authors:** Elaheh Malakan Rad, Ehsan Aghaei‐Moghadam, Mohammad Reza Mirzaaghayan, Hamid Reza Pouraliakbar

**Affiliations:** ^1^ Children's Medical Hospital Center (Pediatric Center of Excellence) Tehran University of Medical Sciences Tehran Iran; ^2^ Rajaie Cardiovascular, Medical & Research Center Iran Univeristy of Medical Sciences Tehran Iran

**Keywords:** ALCAPA, contrast echocardiography, oxygen, pulmonary artery

## Abstract

Pulmonary artery‐focused agitated saline contrast echocardiography unveils tricky cases of ALCAPA by the entry of microbubbles into the left coronary artery (LCA) during systole and retrograde flow from LCA into the main pulmonary artery during diastole. Associated pulmonary hypertension, if present, augments the former flow and supplemental oxygen increases the latter.

## 1 INTRODUCTION

An anomalous left coronary artery from the pulmonary artery (ALCAPA) or Bland‐White‐Garland (BWG) syndrome is a rare and serious congenital heart disease that can lead to sudden cardiac death or dilated cardiomyopathy if not diagnosed timely.^1^, ^2^ Use of all the imaging modalities including echocardiography, coronary computed tomographic angiography, cardiovascular magnetic resonance imaging, and cardiac catheterization and angiography has been reported for detection of this anomaly.^3^, ^4^, ^5^, ^6^ In some cases with the particularly atypical origin and course of the anomalous LCA, color‐Doppler two‐dimensional echocardiography may be deceptive and misleading.^7^ Using pulmonary artery‐focused contrast echocardiography in an infant with a tricky course of anomalous LCA, we introduce a novel two‐dimensional echocardiographic technique to detect ALCAPA in these challenging cases.

## 2 CASE REPORT

A 6‐month‐ and one‐week‐old infant were referred to Children's Hospital Medical Center with the diagnosis of dilated cardiomyopathy. On physical examination, she had tachypnea and tachycardia, and a grade 3/6 regurgitant systolic murmur was heard over the lower left sternal border. Chest X‐ray (CXR) showed cardiomegaly and pulmonary congestion. There were abnormal Q waves in leads 1, aVL, V5, and V6 on the electrocardiogram.

Echocardiographic examination showed left atrial and left ventricular enlargement, severe mitral regurgitation, left ventricular ejection fraction of 30%, hyperechogenic papillary muscles, and prominent flow in the septal perforators (Movies S1 and S2).In the parasternal short‐axis view, both coronary arteries seemed to arise normally from the aorta on two‐dimensional and color‐Doppler echocardiography. Coronary computed tomographic (CT) angiography was performed using a 384‐multislice scanner. The coronary arteries were reported to be normal.

**Figure 1:**
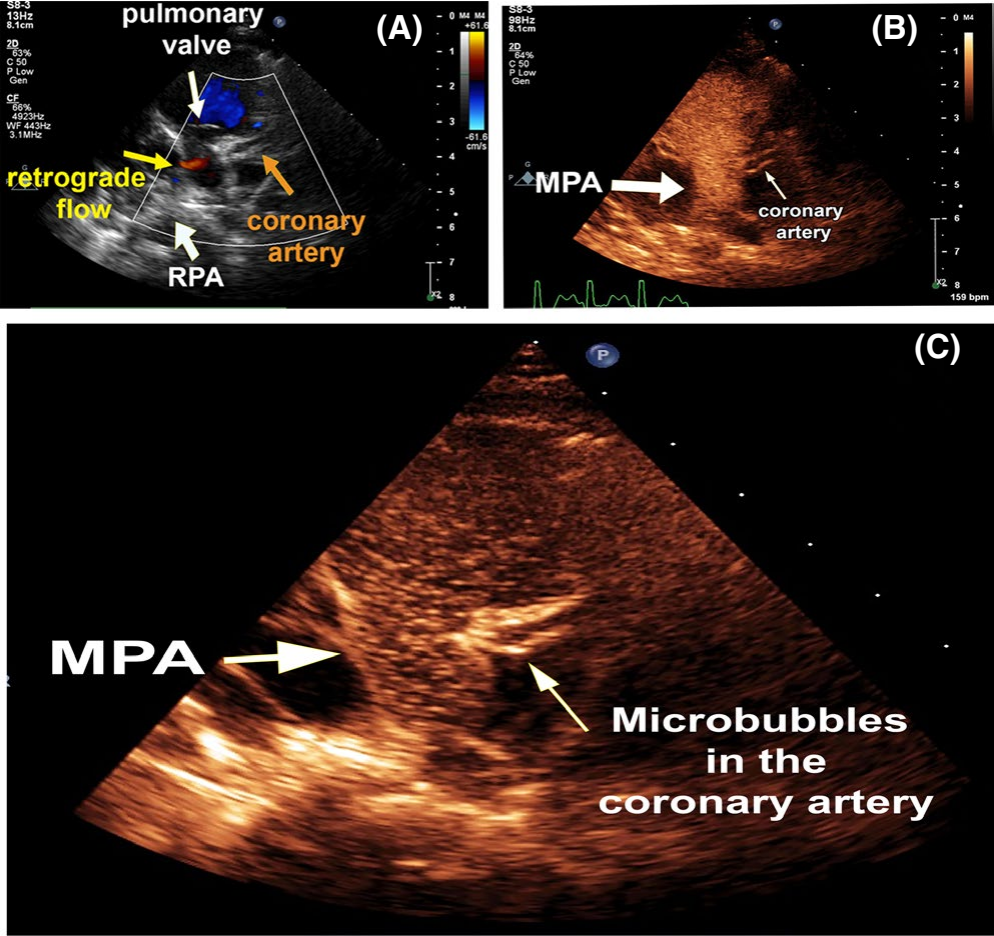
Transthoracic two‐dimensional echocardiography. A, parasternal short‐axis view shows pulmonary valve (white arrow), main pulmonary artery, some segments of the anomalous left coronary artery (orange arrow), and the retrograde flow from the orifice of the anomalous left coronary artery (red flow) characterized by the yellow arrow. B, parasternal short‐axis view, with turning on of the color map for better visualization, indicates the main pulmonary artery is filled with microbubbles of the agitated normal saline. C, microbubbles are seen within the lumen of the anomalous left coronary artery. RPA = right pulmonary artery, MPA = main pulmonary artery

**Figure 2:**
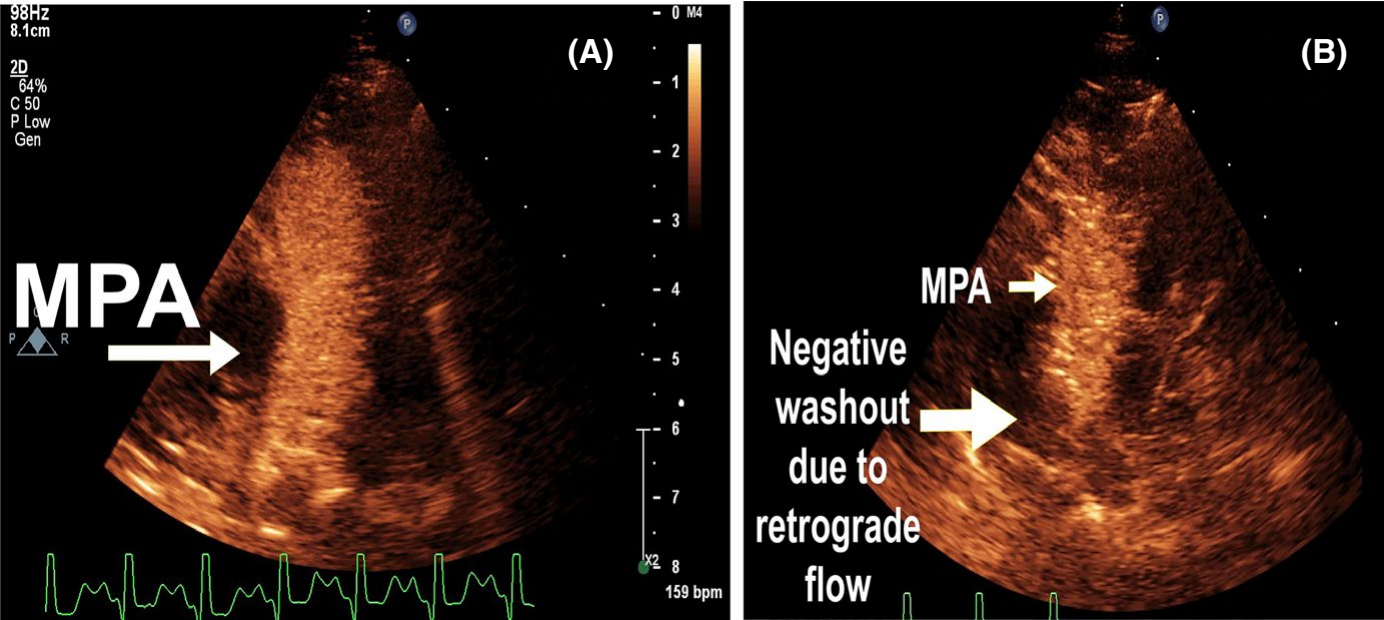
Pulmonary contrast echocardiography. A, Indicates main pulmonary artery filled with microbubbles in the parasternal short‐axis view (color map is turned on for better visualization). B, Shows the negative washout at the location of origin of the anomalous left coronary artery. MPA = main pulmonary artery

**Figure 3:**
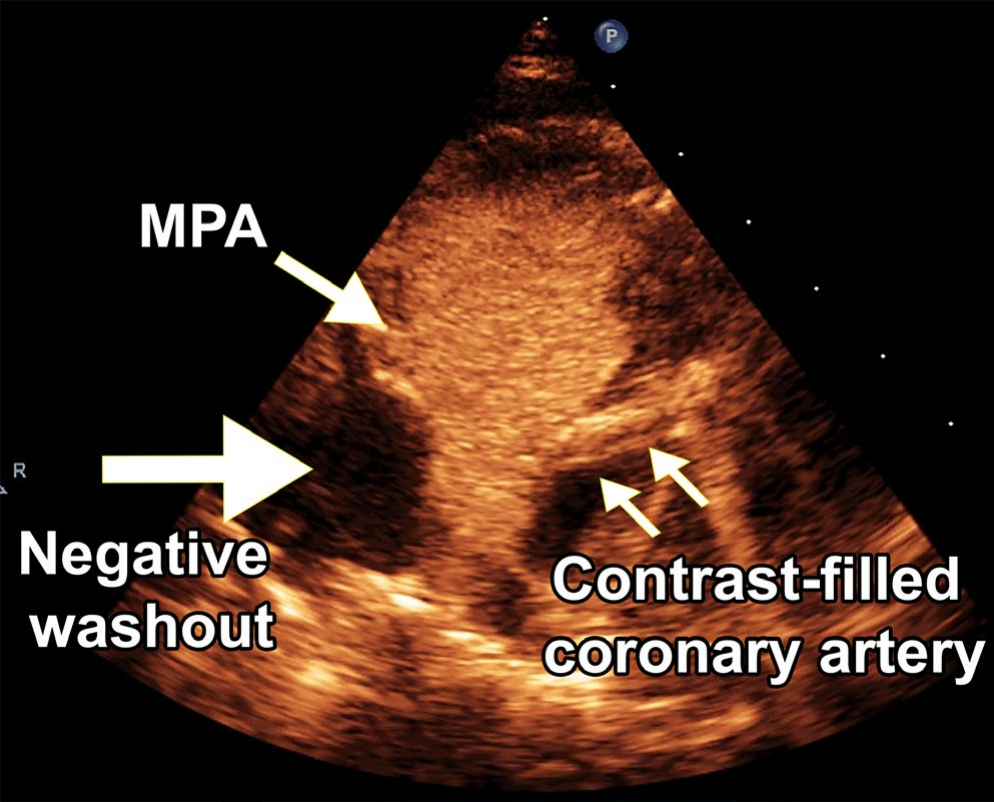
Pulmonary contrast echocardiography. Indicates main pulmonary artery filled with microbubbles in the parasternal short‐axis view (color map is turned on for better visualization). The anomalous left coronary artery is filled with microbubbles (the two smaller white arrows), and the negative washout is also visible at the site of origin of the anomalous LCA, near the origin of the right pulmonary artery (the large white arrow on the left of the Figure). MPA = main pulmonary artery

Since there were several echocardiographic and electrocardiographic findings in favor of anomalous origin of the left coronary artery from the pulmonary artery(ALCAPA), we decided to perform pulmonary contrast echocardiography through injection of agitated saline from the vein on the hand. Firstly, we expected to observe the entrance of the microbubbles into the anomalous left coronary artery after the entry of the microbubbles into the main pulmonary artery (MPA). However, since the child did not have pulmonary hypertension, we also expected to demonstrate the negative washout of flow into the MPA at the origin of the anomalous LCA, due to retrograde flow from the right coronary artery (RCA) via the collateral arteries. To increase the retrograde flow into the pulmonary artery (PA) by transiently decreasing the pulmonary artery pressure, we administered supplemental oxygen with hood for 15 minutes at a flow rate of 10 liters/minute, as described earlier.^8^ We monitored pulse oximetry, electrocardiogram, and blood pressure of the patient. We performed pulmonary contrast echocardiography (PCE) before and after administration of supplemental oxygen (PCESO). After injection of the agitated saline into the left hand, we focused our image on the MPA in the parasternal short‐axis view. After the entrance of the microbubbles into the PA, the anomalous left coronary artery was opacified with microbubbles. Negative washout into the pulmonary artery at the site of origin of the anomalous coronary artery, which was due to retrograde flow through the collateral arteries, was also evident, particularly after administration of supplemental oxygen (Figures 1, 2, 3, Movies S3, S4, S5).

**Figure 4:**
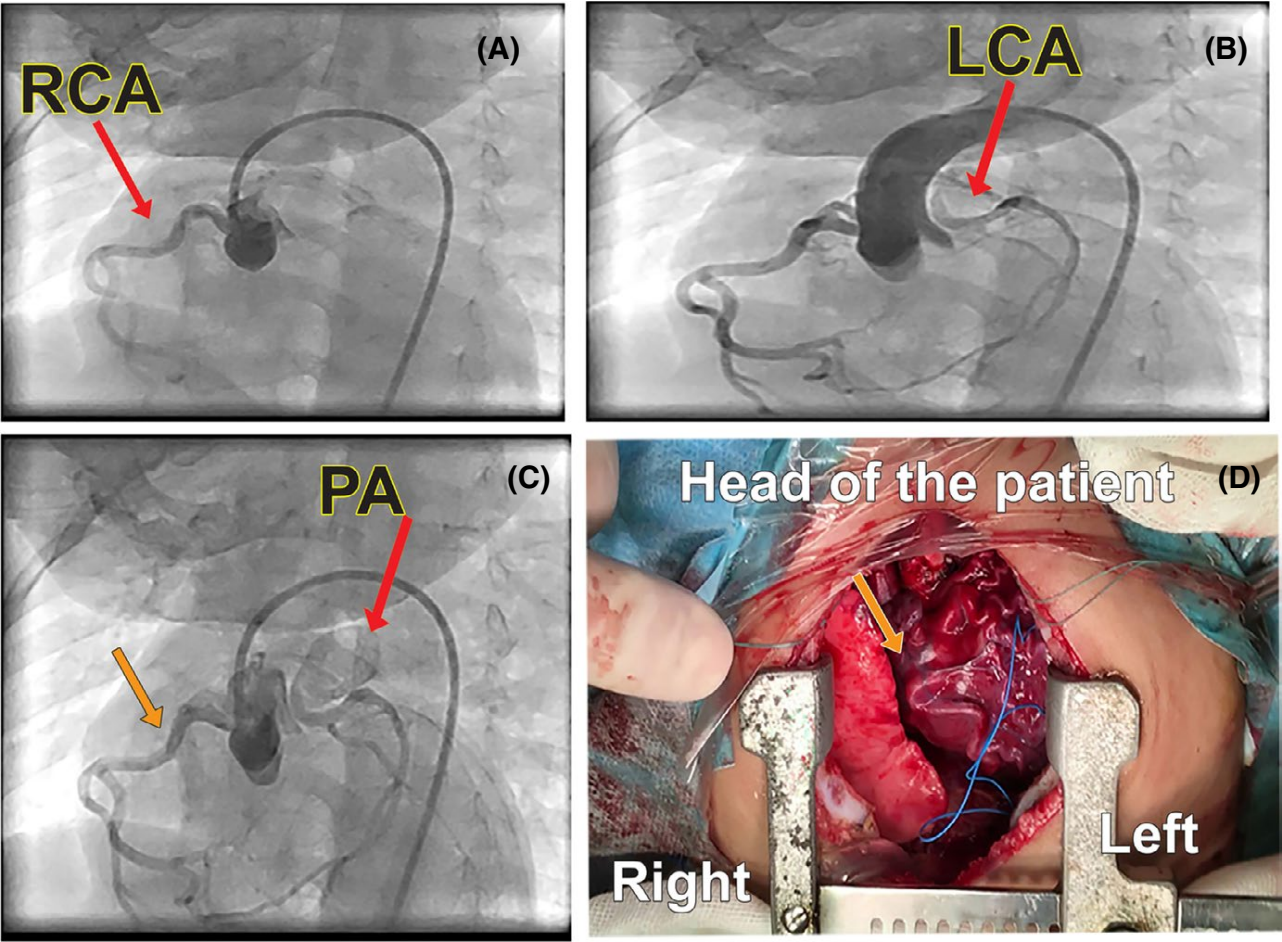
A to C indicates aortogram in 50° left anterior oblique with 30° cranial angulation, in order of the time elapsed after injection of the contrast media. In A, left coronary artery is not visualized, but in B and C, it becomes apparent with a delay. It arises from the pulmonary artery and is filled retrograde by the collateral flow from the right coronary artery. D. The photograph taken in the operation room shows the enlarged right coronary artery and the collateral arteries on the surface of the heart. This photo is 180° rotated clockwise to align with the adjacent Figure of aortogram (ie Figure 4C). Orange arrows show the right coronary artery on Figure 4C,4. PA = pulmonary artery, RCA = right coronary artery, LCA = left coronary artery

Before scheduling for cardiac surgery, we performed cardiac catheterization and angiography under general anesthesia to confirm the diagnosis. Pulmonary artery pressure was 38/20 mm Hg (mean: 28 mm Hg). Aortography showed delayed back‐filling of the anomalous LCA from the pulmonary artery with a high take‐off near the orifice of the right pulmonary artery(RPA). This was best seen in 50° left anterior oblique with 30° cranial angulation (Figure 4, Movie S6).

**Figure 5:**
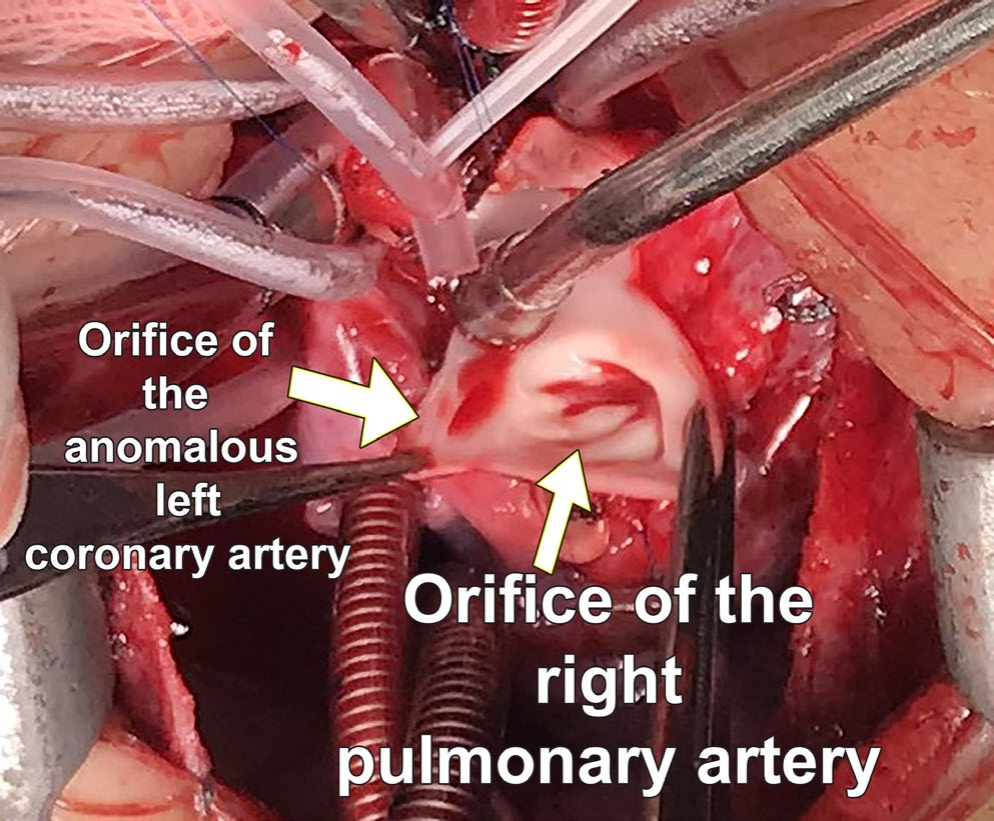
This photograph is taken in the operation room. It indicates the proximity of the orifices of the anomalous left coronary artery and the right pulmonary artery

At operation, there were multiple large collateral arteries on the surface of the right ventricular body and outflow tract. The orifice of the LCA was adjacent to the orifice of RPA (Figure 5), and its wall was fused to the aortic wall. The anomalous LCA was harvested from the pulmonary artery and anastomosed into the left coronary sinus of the aorta. The left coronary sinus of the aorta and the pulmonary artery were reconstructed using the autologous pericardial patch. The patient was discharged in good clinical condition after 1 week. At follow‐up, the left ventricular systolic function had improved, and there was no obstruction in the course of the anastomosed LCA.

**Figure 6:**
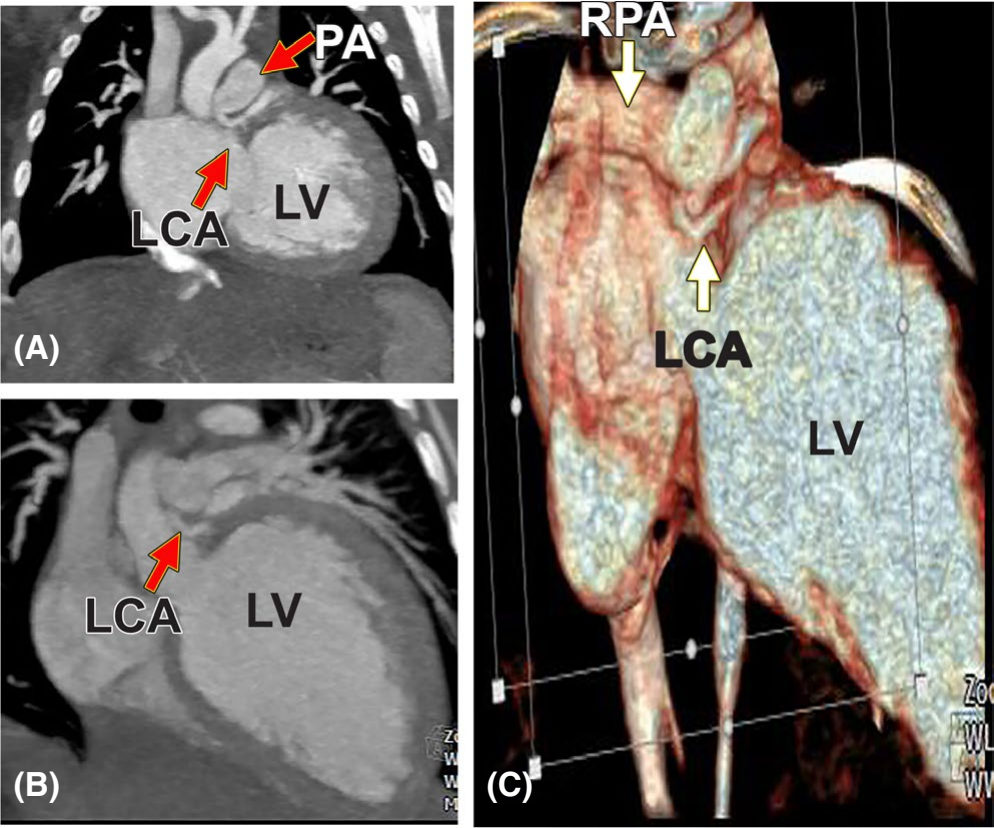
384‐slice coronary computed tomography angiography. A and B show the origin of the anomalous left coronary artery. C, this volume‐rendered image indicates that the orifice of the anomalous left coronary artery is in the proximal right pulmonary artery. PA = pulmonary artery, RPA = right pulmonary artery, LV = left ventricle, LCA = left coronary artery

Using the information obtained at the surgery on the origin and course of the anomalous LCA, retrospective reanalysis of the CT images revealed the anatomy of the anomalous LCA after the operation (Figure 6).

## 3 DISCUSSION

ALCAPA is a congenital anomaly of the coronary arteries which can have a complete cure if diagnosed and repaired timely. However, due to limitations of the echocardiographic machines in providing optimal lateral resolution and the resultant dropout between the wall of the aorta and the wall of the LCA in the parasternal short‐axis view, echocardiography is not infrequently a source of missed diagnosis. There are several subtypes for ALCAPA.^9^ Among these, anomalous origin of LCA from the right pulmonary artery is extremely rare.^10^ Most reported cases of this type are associated with congenital heart diseases such as ventricular septal defect, aortopulmonary window, coarctation, hypoplastic left heart syndrome, and Shone's syndrome.^11^, ^12^, ^13^, ^14^, ^15^ However, our patient had no associated congenital heart disease. Contrast echocardiography is an old technique with increasing applications.^16^ We introduced a novel use of this old technique.

Although cardiac magnetic resonance imaging has been used with success in establishing the diagnosis of ALCAPA, the young age of our patient, and the need for general anesthesia prevented us from using this imaging modality.^5^, ^6^, ^17^

In cases with ALCAPA, either with normal pulmonary arterial pressure or with pulmonary hypertension, pulmonary contrast echocardiography (PCE) with and without prior administration of supplemental oxygen can confirm the diagnosis of ALCAPA by visualization of the following two findings:

1. Antegrade flow from the pulmonary artery into the left coronary artery, which results in opacification of the anomalous LCA with microbubbles after the entrance of the microbubbles into the pulmonary trunk during systole. This flow is seen better in patients with ALCAPA and pulmonary hypertension (PH) during PCE without administration of supplemental oxygen.
2. Retrograde flow from the left coronary artery into the pulmonary artery, which is seen as negative washout into the pulmonary artery, during diastole, at the ostium of the anomalous LCA. This washout is due to the retrograde flow through the collateral arteries, which arise from the RCA. This negative washout flow can be enhanced by lowering pulmonary vascular resistance and pressure through the administration of 100% oxygen during PCE.

The negative washout into the pulmonary artery is not expected to be present in patients with ALCAPA, significant pulmonary hypertension, and negligible collateral arteries.^18^

In this case, antegrade flow from PA into the anomalous LCA occurred in systole and retrograde flow from the LCA into the PA were seen in diastole. Antegrade flow from PA into LCA not only does not increase after oxygen administration but also may decrease. Whereas, oxygen administration augments the retrograde flow from LCA into PA, through a decrease in pulmonary vascular resistance and pressure.

It is noteworthy that in the presence of extensive collaterals and low pulmonary artery pressure, oxygen administration may be hazardous and may result in coronary steal. Therefore, extreme caution should be applied. Short period of oxygen administration, monitoring of the patient's pulse oximetry, electrocardiogram, and blood pressure with meticulous attention to the development of any new ST segment and/or T wave change or changes on the electrocardiogram are necessary. Administration of oxygen must be stopped immediately upon development of any new ST‐T changes, fall in oxygen saturation or blood pressure or deterioration of the patient's clinical condition.

Of note, in the presence of congenital or acquired coronary fistula to the pulmonary artery, we also may expect opacification of the coronary artery after opacification of the pulmonary artery. In summary, with adherence to the above‐mentioned precautions, pulmonary contrast echocardiography with supplemental oxygen (PCESO) is a simple, safe, and almost definitive diagnostic method in patients who are suspected of having ALCAPA. Pulmonary contrast echocardiography may also help unveil the diagnosis in other conditions such as coronary fistula to the pulmonary artery ( as negative washout in the pulmonary artery) and suspicious aortopulmonary window and associated pulmonary hypertension, as the entrance of microbubbles into the aorta (Movie S7). Of course, in the latter case, the absence of atrial septal defect and the ventricular septal defect is necessary. Otherwise, the microbubbles may enter into the aorta not only through the aortopulmonary window but also from the left ventricle, and this may lead to confusion.

## CONFLICT OF INTEREST

None of the authors has any conflict of interest to declare.

## AUTHOR CONTRIBUTIONS

EMR: Developed the concept and design, performed the pulmonary artery‐focused contrast echocardiography of the patient, drafted the article, critically revised the manuscript for important intellectual content, and approved the final version. EA: Performed the clinical management of the patient, critically revised the manuscript for important intellectual content, and approved the final version. MRM: Performed the cardiac surgery of the patient, provided postoperative care, critically revised the manuscript for important intellectual content, and approved the final version. HP: Performed and interpreted the computed tomographic angiographies of the patient, critically revised the manuscript for important intellectual content, and approved the final version.
